# Comparative proteomics study on liver mitochondria of primary biliary cirrhosis mouse model

**DOI:** 10.1186/1471-230X-13-64

**Published:** 2013-04-12

**Authors:** Guang Song, Chaojun Hu, Huishan Zhu, Xi Li, Liying Zhao, Renfang Zhou, Xuan Zhang, Fengchun Zhang, Lin Wu, Yongzhe Li

**Affiliations:** 1 Department of Rheumatology and Clinical Immunology, Peking Union Medical College Hospital, Peking Union Medical College, Chinese Academy of Medical Sciences, Beijing 100032, PR China; 2 Beijing Institute of Genomics, Chinese Academy of Sciences, Beijing 100029, PR China; 3 Beijing Protein Innovation Ltd. Co, Beijing 101318, PR China

**Keywords:** Primary biliary cirrhosis, Proteomics, iTRAQ, Molecular pathogenesis

## Abstract

**Background:**

Primary biliary cirrhosis (PBC) is a liver specific chronic disease with unclear pathogenesis, especially for the early stage molecular events. The mitochondrion is a multi-functional organelle associated with various diseases including PBC. The purpose of this study was to discover the alterations in the mitochondria proteome using an early stage PBC mouse model for revealing the possible pathogenesis mechanisms in the early stages of PBC.

**Methods:**

Mouse model of early stage of PBC was constructed by consecutive administration of poly I:C. Mitochondria of mouse models and controls were purified and comparative proteomics was performed by iTRAQ technology. Then, differentially expressed proteins were validated by western blotting.

**Results:**

In total 354 proteins that satisfied the criteria for comparative proteomics study were identified. Of them, nine proteins were downregulated and 20 were up-regulated in liver mitochondria of PBC mouse model. Most differentially expressed proteins are associated with oxidation-reduction and lipid metabolism, and some are involved in the biosynthesis of steroid hormone and primary bile acid. Interestingly, four proteins (HCDH, CPT I, DECR, ECHDC2) involved in the fatty acid beta-oxidation were all upregulated.

**Conclusions:**

iTRAQ is a powerful tool for comparative proteomics study of PBC mouse model and differentially expressed proteins in mitochondria proteome of PBC mouse model provide insights for the pathogenesis mechanism at early stage of PBC.

## Background

Primary biliary cirrhosis (PBC) is a chronic inflammatory liver disease characterized by progressive destruction of small and medium-sized bile ducts, which leads to cholestasis, liver fibrosis, cirrhosis, and ultimate liver failure [1,2]. It primarily affects middle-aged women, with the ratio of female/male patients at about 8–10:1 [3,4]. PBC is considered as an autoimmune disease and the serologic hallmark is high titers of anti-mitochondria antibodies (AMA), whose main target is the lipoyl domain of the E2 subunits of the 2-oxo-acid dehydrogenase complexes located at mitochondrial inner membrane.

PBC progresses through four histological stages according to the guidelines approved by the American Association for the Study of Liver Diseases (AASLD). Stage I is characterized by portal inflammation with or without florid bile duct lesions and the inflammation is restricted to portal area. At stage II, periportal lesions extend into the hepatic parenchyma. Stage III is characterized by a distortion of the hepatic architecture with numerous fibrous septa. Cirrhosis with the existence of regenerative nodules defines stage IV [5].

PBC is thought to be triggered by combination of multiple genetic and environmental factors, but the molecular pathogenesis is still enigmatic, especially for cellular process at early stages, which might be important for discovery of the Pandora box of PBC. Through the literature search, we found mitochondria might be involved in the pathogenesis of PBC. First, in vitro treatment of poly I:C, a synthetic analog of viral double-stranded RNA (dsRNA), induced mitochondrial dysfunction in cultured human hepatocytes [6]. Second, in the condition of cholestasis, the retention of hydrophobic bile acids can induce oxidative damage, causing mitochondrial dysfunction, and induce endoplasmic reticulum stress, which were involved in triggering apoptosis of hepatocyte and biliary epithelial cells [7]. Furthermore, mitochondria had been confirmed to be involved in the processing of liver fibrosis in diseases of viral hepatitis [8], alcoholic and nonalcoholic steatohepatitis [9], drug-induced liver injury [10], and so on. A close look at proteome level alterations in liver mitochondrial proteins might reveal some insights for PBC pathogenesis.

However, it is difficult to carry out the molecular research at early stage of PBC, as for most patients present with clinical symtoms in the later stages. Mouse models have been served as powerful tools in the pathogenesis research of various diseases. Okada et al. reported that consecutive administration of Poly I:C to C57BL/C induced PBC like lesions and serological AMA similar to features of PBC [11], and was considered as mouse model for research at the early stage of PBC [12-14].

As an initial step to reveal the pathogenesis development of PBC, in this study, we prepared PBC mouse model by consecutive injection of poly I:C to C57BL/6 mice and quantitatively compared liver mitochondria proteome between model and control groups by iTRAQ technique. Gene ontology analysis was performed to classify the differentially expressed proteins. At last, some differentially expressed proteins identified by iTRAQ were validated by western blotting.

## Methods

### Mouse model

Mouse model was constructed according to the method described previously [11,13] with minor modification. Adult 6–8 week-old C57BL/6 female mice were injected intraperitoneally twice a week with poly I:C (Sigma-Aldrich, St. Louis, USA) (PBC model group) at a dose of 5 mg/kg body weight or with the same volume of saline water (control group). Mice were sacrificed by cervical dislocation at three time points: four, eight, and 12 weeks after initiation of injection. Livers were collected and washed three times with saline water. Each liver was cut into two pieces. The small piece was immediately fixed in buffered formalin for pathological staining, and the large piece was subjected to mitochondria purification. All mice were maintained in a pathogen-free facility at Institute of Basic Medical Sciences Chinese Academy of Medical Sciences (Beijing, China) and received humane care before sacrifice.

### Histopathological analysis

Liver samples were fixed in formalin solution and then embedded in paraffin. Subsequently, the embedded liver tissue was cut into thin slices. After deparaffinization, liver slices on the slides were stained with hematoxylin and eosin (H&E). The Infiltration of Inflammatory cells in the liver tissues was evaluated with light microscopy by senior pathologist at Beijing Union Medical College Hospital [5].

### Purification of mitochondria

Mitochondria purification was conducted following the method described previously [15]. In short, liver samples were minced and homogenized in pre-cooled homogenization buffer (0.25 M sucrose, 10 mM HEPES pH 7.5, 1 mM EDTA, 0.5 mM EGTA, 1 mM PMSF, 1 mM NaF and 0.2 mM Na_3_VO_4_). Crude mitochondria were enriched by differential centrifugation, and were further purified by centrifugation in a 20-34% Nycodenz density gradient at 52,000 g for 90 min. Mitochondria fraction was collected at the interface of 25/30% density and resuspended in mitochondrial resuspending buffer (200 mM mannitol, 50 mM sucrose, 10 mM Tris–HCl, pH 7.4, 1 mM EDTA, 0.5 mM EGTA, 1 mM PMSF, 1 mM NaF and 0.2 mM Na_3_VO_4_). An additional centrifugation at 20,000 g for 30 min was carried out to get the final purified mitochondria pellet. The pellets were stored at-80°C until use. The purity of the mitochondria were evaluated by western blotting using mitochondria specific antibody, anti-ATPase subunit beta (ATPB) antibody (Santa Cruz, CA, USA), and cytoplasm specific antibody, anti-ALD antibody (Beijing protein innovation Ltd., Co., Beijing, China).

### Mitochondria protein preparation and trypsin digestion

Total mitochondria proteins were prepared by dissolving the purified mitochondria pellet into lysis buffer (7 M urea, 2 M thiourea, 4% CHAPS, 1 mM PMSF, 2 mM EDTA and 40 mM Tris–HCl). After sonication and centrifugation at 20,000 g for 10 min, the supernatant were collected as mitochondrial proteome solution. Protein concentration was measured using Bradford method.

Equal amount of mitochondria protein from four mice of the same group at 12-week time point were pooled for further analysis. After reduction and alkylation, pooled mitochondrial proteins were precipitated by pre-cooled acetone and resolved by 50% Tetraethylammonium Bromide (TEAB) (Sigma-Aldrich, St. Louis, USA), and then 150ug protein samples were digested into peptides by sequence grade trypsin (Promega, Madison, WI) for 36 h at 37°C before stopped by 0.1% formic acid (FA). At last, the digested samples were dried in a centrifugal vacuum concentrator.

### Peptide labeling and separation

Peptide pellets from mitochondrial protein of model and control groups were individually dissolved in 30ul of 50% TEAB with 70 μL isopropanol, and then labeled with iTRAQ reagents 113, and 115, respectively, according to the manual of 8-plex iTRAQ labeling kit (Applied Biosystems, Foster City, CA, USA). The labeling efficiency for each sample was 98.0%. The labeled peptides from two groups were mixed together and unbound iTRAQ reagents were removed through Strata™-X-C (Phenomenex Inc., Torrance, CA, USA). Subsequently, the labeled peptides were separated based on their isoelectric points (pI) by isoelectric focusing (IEF) on a Multiphor II unit (GE Healthcare, Fairfield, CT, USA) using an Immobiline Drystrip Gradient (IPG) pH 3–10 gel (GE Healthcare, Fairfield, CT, USA). Then, the strips were cut into 44 equal pieces along pH gradient, and each piece was consecutively incubated in 100ul of 0.1% FA, 0.1%FA/50% Acetonitrile (ACN), and 0.1%FA/80%ACN for 10 min each to extract labeled peptides from gel. The extractions from the same gel piece were combined as one fraction and dried in a centrifugal vacuum concentrator. Each fraction was desalted using Strata™-X column (Phenomenex Inc., Torrance, CA, USA) according to its manual. The samples were then dried using a vacuum centrifuge followed by resuspension in 30ul 0.1% (v/v) formic acid. The relative amount of peptides in each fraction was estimated by sampling with MALDI-TOF (Bruker Daltonics Inc, Waltham, USA), and some adjacent fractions with lower signals were pooled and dried using a vacuum centrifuge and then re-dissolved in 20ul 0.1% (v/v) formic acid.

### ESI-Q-TOF analysis

Analysis of peptides was processed by Quadrupole-Time of Flight Mass spectrometer, MicroTOF-Q II (Bruker Daltonics Inc, Waltham, USA), after reverse phase liquid chromatography. Firstly, 10ul of peptides in each fraction were subjected to liquid chromatography (Proxeon Easy nano-LC, Odense, Denmark) on a C18 reverse phase column (100 mm × 75um) for 120 min at a flow of 0.3ul/min of a mixture of two solvents. Solvent A was water/0.1% formic acid, and solvent B was ACN/0.1% formic acid. The percentage of solvent B to the total flow was changed to 5%, 45%, 80% and 5% at 0 min, 10 min, 80 min, and 100 min point, respectively. Mass spectra were performed in a data dependent mode in which three most abundant ions for each MS scan were selected for MS/MS analysis within 50-3000 m/z scan range. Trypsin autolysis products and keratin-derived precursor ions were automatically excluded. Raw data files were converted into Mascot Generic Format (MGF) files with Data Analysis Software (Bruker Daltonics Inc, Waltham, USA) and then merged into a single MGF file for all fractions.

### Database searching

MS/MS data searching was processed with Mascot software (Matrix Science, London, UK; version Mascot). Mascot was set up to search the ipi.MOUSEv3.79 database assuming the digestion enzyme trypsin, and then searched with a fragment ion mass tolerance of 0.05 Da and a parent ion tolerance of 0.05 Da. Iodoacetamide derivative of cysteine was specified in Mascot as a fixed modification. S-carbamoylmethylcysteine cyclization (N-terminus) of the n-terminus, oxidation of methionine and iTRAQ-8plex of lysine, tyrosine and the n-terminus were specified in Mascot as variable modifications.

### Protein identification and quantitative analysis

In order to make the quantification more accurate, Scaffold (version Scaffold_3.3.1, Proteome Software Inc., Portland, OR) was used to validate MS/MS based peptide and protein identifications resulted from Mascot search. Peptide identifications were accepted if they could be established at greater than 95.0% probability as specified by the Peptide Prophet algorithm [16] (Keller, A 2002). Protein identifications were accepted if they could be established at greater than 99.0% probability and contained at least 2 identified unipeptides. Protein probabilities were assigned by the Protein Prophet algorithm [17]. Proteins that contained similar peptides and could not be differentiated based on MS/MS analysis alone were grouped to satisfy the principles of parsimony. In this study, the false discover rates (FDR) of protein identification at both peptide and protein levels were 0.0%.

Peptides met the above criteria were quantified using the centroided reporter ion peak intensity. Intra-sample channels were normalized based on the median ratio for each channel across all proteins. Multiple isobaric tag samples were normalized by comparing the median protein ratios for the reference channel. Protein quantitative values were derived only from uniquely assigned peptides. The minimum quantitative value for each spectrum was calculated as the 5.0% of the highest peak. Protein quantitative ratios were calculated as the median of all peptide ratios. Due to limited quantity of mitochondria samples, no technical replication was performed and so we set the criteria of fold change >1.4 or <0.72 between PBC mouse model and control for identifying differentially expressed proteins, more stringent than that accepted by several previous research [18-22], in which the cutoff were at >1.2 or <0.82. Data were further analyzed for protein subcellular location and functional cluster using Scaffold software based on NCBI online database and UniProt database, respectively.

### Western blotting

Individual or pooled mitochondria protein samples (5 ug) were loaded for 12% sodium dodecyl sulfate polyacrylamide gel electrophoresis (SDS-PAGE) and gel separated proteins were transferred to Polyvinylidene fluoride (PVDF) membranes (Millipore, MA USA). The membranes were blocked with 5% skimmed milk dissolved in PBS with 0.1% Tween20 and then incubated with 1:2,000 diluted primary antibodies generated in rabbits (Beijing Protein Innovation Ltd., Co. Beijing, China) at 37°C for 1 h. After washed with PBST for 10 min for three times, the membranes were incubated with horseradish peroxidase (HRP) labeled sheep anti-rabbit antibody (diluted 1:10,000) for 1.5 h at room temperature. After rinsing the membrane with PBST, the immune-recognition images from chemical-luminescent signals using the ECL kit (Beijing Applygen Ltd.,Co., Beijing, China) were visualized by ImageQuant RT ECL (GE Healthcare, Fairfield, CT, USA).

## Results

### Preparation of PBC mouse model

**Figure 1:**
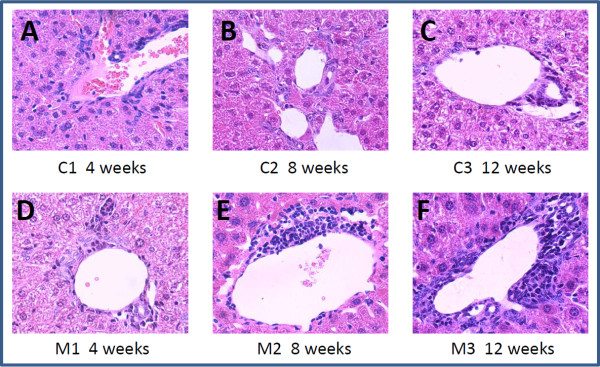
**HE staining results of liver tissues from PBC mouse models and controls at various time points. A**, **B** and **C** are the staining images from control groups at 4, 8, and 12 weeks, respectively, showing no abnormal alteration. While **D**, **E** and **F** are the staining images from the PBC mouse model group, showing that inflammatory cells infiltrated around the portal areas (all images are in × 400 magnification).

**Figure 2:**
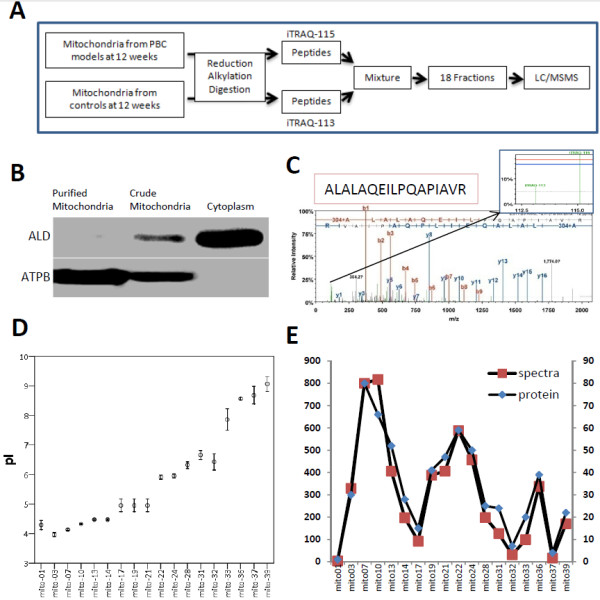
**Procedure of quantitative iTRAQ proteomics.** (**A**) Strategy for comparative proteomics study on the liver mitochondria between mouse models and controls. (**B**) Evaluation of mitochondrial purity with organelle specific antibodies. (**C**) An example of the spectra, showing peptide (ALALAQEILPQAPIAVR) which is one of the three unipeptides for Isoform 1 of Enoyl-CoA hydratase domain-containing protein 2 (ECHDC2). (**D**) Distribution of peptides in each fraction according to their isoelectric points. Error bars show 95% confidence interval. (**E**) Quantities of peptide spectra and proteins in each fraction.

To study the proteome change in liver mitochondria at early stage of PBC, we first prepared PBC mouse model by consecutive injection of poly I:C to C57BL/6 mice. We collected liver samples at the end of four, eight, and 12 weeks, because during these time points the mice would be in the early stage of PBC [11-13]. Histopathological analysis was performed to evaluate the progress of the disease (Figure 1). As previously reported, with four weeks of poly I:C injection, mice had already exhibited PBC-like lesions at portal area, a characteristic of stage I of PBC according to the guideline approved by AASLD [5]. With eight and 12 weeks of injection, the lesions were still limited in the portal area, but the inflammatory cell infiltration was aggravated with increased numbers of injections. Meanwhile, all mice in the control group had none PBC-like lesions. We chose week 12 for comparative proteomics study by iTRAQ and the strategy is shown in Figure 2A.

### Mitochondria sample preparation

To identify the differentially expressed proteins in mitochondria of PBC mouse models, we purified mitochondria from each mouse liver, extracted total proteins and combined equal amount from the same group at 12-week time point. The purity of mitochondria were demonstrated by western blotting of total mitochondria proteins and cytoplasm proteins using anti-ATP synthesis subunit B (ATPase subunit B) and anti-Aldehyde reductase (ALDR) antibodies (Figure 2B), target of the former is specific to mitochondrial and the later is to cytoplasm. The unique band recognized by anti-ATPase subunit B antibody appeared in the purified and crude mitochondrial samples but not in the cytoplasm fractions, while anti-ALDR antibody did not recognize any proteins in purified mitochondrial samples, indicating the purified mitochondria were in good quality.

### Comparative proteomics analysis by iTRAQ

To examine the difference in liver mitochondria proteome between PBC mouse models and controls, we conducted the analysis by iTRAQ. After trypsin digestion of total mitochondrial proteins, peptides from the model and control groups were labeled with 115 and 113, respectively. We then mixed the two samples, separated the peptides based on isoelectric points by IEF on a linear pH 3–10 strip, and extracted peptides from 44 consecutive pieces along pH gradient. To save the machine time, we first checked the peptide quality and abundance in each fraction by MALDI-TOF, and combined the adjacent fractions with little peptides, resulting in a total of 18 fractions for ESI-Q-TOF detection (Figure 2). Figure 2C shows a representative peptide spectrum of ESI-Q-TOF detection. Figure 2D shows the isoelectric point distribution of peptides identified in each of the 18 fractions, clearly demonstrating the well separation of peptides by IEF. Figure 2E shows the number of peptides and proteins identified in each fraction. The majority of peptides were distributed within the pI ranges of 4.1-5.4, 5.92-6.49, and 7.71-8.26, the result was similar to previously reported [23].

The MGF file merged from all the 18 fractions was subjected to database searching by Mascot software. In total, we identified 648 proteins. In order to make the quantification more accurate, we performed protein identification and quantification with Scaffold software using following criteria: 1) Peptide identification was more than 95% probability; 2) Protein identification was greater than 99% probability; 3) At least two unique peptides were identified for each protein. A total of 8978 spectra for 354 proteins satisfied the criteria. According to the sub-cellular localization information from NCBI, 68.4% (242/354) proteins are annotated mitochondrial proteins.

To analyze the quantitative data, we calculated ratios of peptides with the median of corresponding spectra and ratios of proteins with the median of corresponding peptides. With a threshold at over 1.4 fold, 29 proteins were identified as differentially expressed proteins between mouse models and controls at 12-week time point. Of these, nine were down-regulated (Table 1, Additional file 1: Table S1) and 20 were up-regulated in liver mitochondria of PBC model mice (Table 2, Additional file 1: Table S2).

**Table 1:** Down-regulated proteins in liver mitochondria of PBC mouse model

**IPI_ID**	**Uniprot name**	**Protein name**	Ratio*
IPI00119940	CP17A_MOUSE	Steroid 17-alpha-hydroxylase/17,20 lyase	0.54
IPI00225288	CC90B_MOUSE	Isoform 1 of Coiled-coil domain-containing protein 90B	0.54
IPI00127223	B9EIY3_MOUSE	UDP glucuronosyltransferase 2 family, polypeptide B36	0.57
IPI00890309	A2BIN1_MOUSE	major urinary protein 10	0.57
IPI00850133	K0564_MOUSE	Isoform 1 of Uncharacterized protein KIAA0564 homolog	0.66
IPI00119685	CP27A_MOUSE	Sterol 26-hydroxylase, mitochondrial	0.71
IPI00127050	IPYR2_MOUSE	Isoform 1 of Inorganic pyrophosphatase 2, mitochondrial	0.71
IPI00133240	UCRI_MOUSE	Cytochrome b-c1 complex subunit Rieske, mitochondrial	0.71
IPI00316314	HACL1_MOUSE	2-hydroxyacyl-CoA lyase 1	0.71

*Ratio refers to protein ratio of PBC mouse model/control.

**Table 2:** Up-regulated proteins in liver mitochondria of PBC mouse model

**IPI_ID**	**Uniprot name**	**Protein name**	Ratio*
IPI00116222	3HIDH_MOUSE	3-hydroxyisobutyrate dehydrogenase, mitochondrial	1.41
IPI00120212	NDUA9_MOUSE	NADH dehydrogenase [ubiquinone] 1 alpha subcomplex subunit 9,	1.41
IPI00121105	HCDH_MOUSE	Hydroxyacyl-coenzyme A dehydrogenase, mitochondrial	1.41
IPI00126050	PGCP_MOUSE	Isoform 1 of Plasma glutamate carboxypeptidase	1.41
IPI00309073	MTP_MOUSE	Isoform 1 of Microsomal triglyceride transfer protein large subunit	1.41
IPI00319973	PGRC1_MOUSE	Membrane-associated progesterone receptor component 1	1.41
IPI00554961	FMO5_MOUSE	dimethylaniline monooxygenase [N-oxide-forming] 5	1.41
IPI00117914	ARGI1_MOUSE	Arginase-1	1.47
IPI00225390	CX6B1_MOUSE	Cytochrome c oxidase subunit 6B1	1.51
IPI00118986	ATPO_MOUSE	ATP synthase subunit O, mitochondrial	1.52
IPI00123281	LRC59_MOUSE	Leucine-rich repeat-containing protein 59	1.52
IPI00134870	ACOX2_MOUSE	Peroxisomal acyl-coenzyme A oxidase 2	1.52
IPI00307837	EF1A1_MOUSE	Elongation factor 1-alpha 1	1.52
IPI00330094	CPT1A_MOUSE	Carnitine O-palmitoyltransferase 1, liver isoform	1.52
IPI00115598	DHB8_MOUSE	Isoform Short of Estradiol 17-beta-dehydrogenase 8	1.62
IPI00221400	ADH1_MOUSE	Alcohol dehydrogenase 1	1.74
IPI00387379	DECR_MOUSE	2,4-dienoyl-CoA reductase, mitochondrial	1.74
IPI00469195	ECHD2_MOUSE	Isoform 1 of Enoyl-CoA hydratase domain-containing protein 2,	1.75
IPI00757372	ISC2A_MOUSE	Isochorismatase domain-containing protein 2A, mitochondrial	1.87
IPI00169463	TBB2C_MOUSE	Tubulin beta-2C chain	2.14

*Ratio refers to protein ratio of PBC mouse model/control.

### Differentially expressed proteins

In order to gain insight into the biological meaning of the differentially expressed proteins in the liver mitochondria of PBC mouse models versus controls, Gene Ontology (GO) analysis was performed and differential proteins were categorized according to the cellular component, biological process, and molecular function.

**Figure 3:**
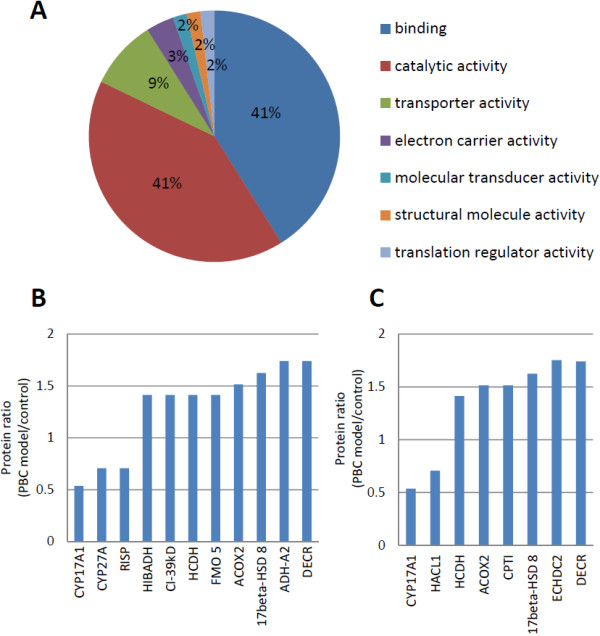
**GO classification of differentially expressed proteins in liver mitochondria between PBC mouse model and controls.** (**A**) GO analysis based on molecular functions. Gene function annotation showed the differential proteins were mainly associated with (**B**) oxidation reduction and (**C**) lipid metabolism.

Based on analysis of the cellular component, 65.3% (17 of the 26 proteins with annotation information) of the differentially expressed proteins were annotated mitochondrial proteins. In the molecular function analysis of GO analysis (Figure 3A), most differentially expressed proteins were associated with function of binding (42%) and catalytic activity (41%).

Classification analysis of the biological processes with UniProt database and DAVID online tool (http://david.abcc.ncifcrf.gov/) showed the differentially expressed proteins are mainly involved in the processes of oxidation reduction and lipid metabolism, including 11 and 8 proteins, respectively (Figure 3B-C). For the oxidation reduction, three proteins were down-regulated and eight were up-regulated in the liver mitochondria of PBC mouse models. The down-regulated proteins were steroid 17-alpha-hydroxylase/17,20 lyase (CYP17A1), sterol 26-hydroxylase (CYP27A), and cytochrome b-c1 complex subunit Rieske (RISP). The up-regulated proteins were 3-hydroxyisobutyrate dehydrogenase (HIBADH), NADH dehydrogenase [ubiquinone] 1 alpha subcomplex subunit 9 (CI-39kD), hydroxyacyl-coenzyme A dehydrogenase (HCDH), dimethylaniline monooxygenase [N-oxide-forming] 5 (FMO 5), peroxisomal acyl-coenzyme A oxidase 2 (ACOX2), isoform short of estradiol 17-beta-dehydrogenase 8 (17 beta-HSD 8), alcohol dehydrogenase 1 (ADH-A2), and 2,4-dienoyl-CoA reductase (DECR). Among those proteins, two are associated with steroid hormone biosynthesis; 17beta-HSD 8 was up-regulated, while CYP17A1 was down-regulated in PBC mouse model. Additionally, CYP27A, involved in the metabolism of primary bile acid biosynthesis, was down-regulated in liver mitochondria of PBC model.

Four of the eight proteins involved in the lipid metabolic process, hydroxyacyl-coenzyme A dehydrogenase (HCDH), carnitine O-palmitoyltransferase 1 (CPT I), 2,4-dienoyl-CoA reductase (DECR), and isoform 1 of Enoyl-CoA hydratase domain-containing protein 2 (ECHDC2), specifically to fatty acid beta-oxidation system, the major degradation pathway for fatty acid in humans. Interestingly, all of the above four proteins were up-regulated in liver mitochondria of PBC mouse models. In contrast, 2-hydroxyacyl-CoA lyase 1 (HACL1) in the fatty acid alpha-oxidation setting was down-regulated.

### Validation of differentially expressed proteins by western blotting

**Figure 4:**
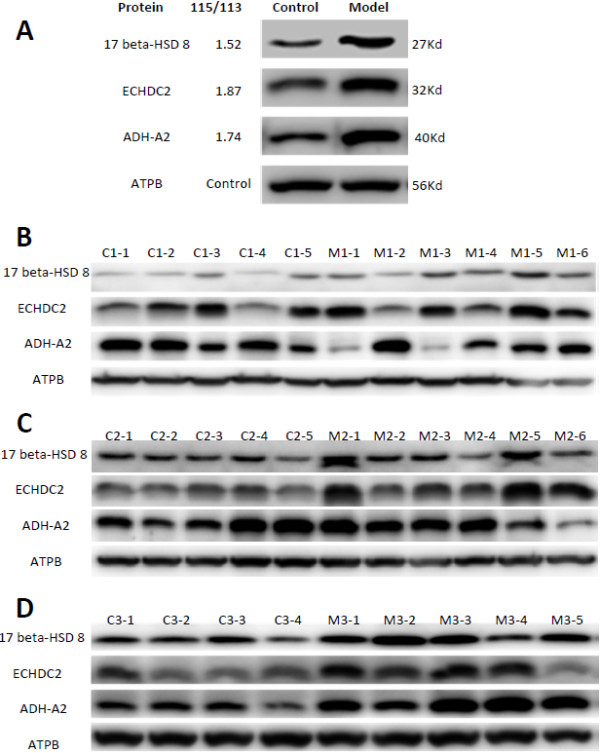
**Validation of three up-regulated mitochondria proteins by western blotting.** Antibodies against isoform short of estradiol 17-beta-dehydrogenase 8 (17 beta-HSD 8), , Isoform 1 of Enoyl-CoA hydratase domain-containing protein 2 (ECHDC2), and alcohol dehydrogenase 1 (ADH-A2) were generated and used in western blotting with pooled mitochondria protein at week 12, the same samples used in iTRAQ (**A**), and samples from individual mouse of both model and control groups at week 4 (**B**), 8 (**C**) and 12 (**D**). ATPB was used as equal loading control marker, the expression of which had no significant difference between PBC model mice and controls (R_115/113_ = 1.1).

To validate the differentially expressed proteins identified by iTRAQ technique, we used western blotting, a semi-quantitative protein technique, to detect the expression of proteins in total liver mitochondria proteins from PBC mouse model and controls. Eight proteins were randomly selected from the differential expressed proteins involved in oxidation reduction and/or lipid metabolism. Peptides containing putative antigenic regions were chemically synthesized and used to prepare polyclonal antibodies in rabbits. At last, three antibodies (against ECHDC2, 17beta-HSD 8, and ADH-A2, respectively) were in good quality, each recognized a single band in total mitochondria protein at expected molecular weight (data not shown). Validation by western blotting was processed using the pooled and individual mitochondrial samples in both PBC mouse model and control groups at different time points (Figure 4). For each protein, the signal intensities of the pooled mitochondria samples from PBC mouse model group at weeks 12 were significantly higher than that from controls, which were consistent with the result from iTRAQ (Figure 4A). For individual mitochondria samples, there are large variations in earlier weeks. Towards the later weeks, it was obvious that the protein levels in PBC models were generally higher than those in controls (Figure 4B-D).

## Discussion

PBC is a liver specific chronic disease with unclear pathogenesis, especially the molecular events at early stage. In this study, we constructed the mouse model of early stage of PBC using poly I:C administration to female C57BL/6, performed the comparative proteomics study of liver mitochondria between PBC model and control mice, and identified several differentially expressed mitochondria proteins.

This PBC mouse model was first reported by Okada et al. [11], who found administration of poly I:C induced PBC like lesions, serologic AMA, and elevated alkaline phosphatase activity, which are the three important diagnostic criteria for the disease. Studies have shown that TLR3 pathway is critical in the pathogenesis of PBC [24-26], and poly I:C, a synthetic double-stranded RNA, can mimic the infection of retroviral RNA by interacting with TLR3 and consequently activating the innate immunity with series of cytokine production and activation of lymphocytes. This mouse model has been repeatedly constructed by a number of laboratories for uncovering the cellular and molecular events in pathogenesis of PBC [12-14]. Similar to others, our model also exhibited lymphocyte infiltration to the portal area as early as 4 weeks after initiation of poly I:C injection, and at the end of 12 weeks the model was still at stage I.

We employed iTRAQ method to analyze protein expression patterns in the liver mitochondria of PBC model. Of all 354 proteins identified, 68.4% (242/354) are annotated mitochondrial proteins, while others either had no localization information or were annotated within other compartments. The identification of proteins annotated outside the target compartment was also seen in other organelle proteomics research [27]. The reason for that may be due to incomplete annotation, strong interaction with the proteins located at outer mitochondrial membrane, or contamination of trace amount of other organelles.

Our comparative proteomics research on the liver mitochondria identified nine down-regulated and 20 up-regulated proteins in PBC mouse model. The differentially expressed proteins are mainly involved in the process of oxidation reduction and lipid metabolism. Among the 11 proteins that are associated with oxidation reduction, one reductase, two oxidases, and five dehydrogenases were significantly up-regulated, which might satisfy a higher demand of energy for the inflammatory response [28] seen early on in the PBC model as the infiltration of the inflammatory cells around bile duct (Figure 1). Additionally, increased alcohol dehydrogenase had been shown to associate with inflammatory response and the Reactive Oxygen Species (ROS) in the development of alcoholic liver disease [29]. In our study, alcohol dehydrogenase is confirmed up-regulated (Figure 4), and it might function in inflammatory response in the development of PBC as well. On the other hand, in the lipid metabolism, all the four proteins associated with beta-oxidation, the classic pathway for the degradation of fatty acid, were up-regulated. While in patients with alcohol- and obesity-induced fatty liver diseases, impaired mitochondrial fatty acid beta-oxidation plays a key role in liver steatosis, fibrosis, and eventually cirrhosis [30,31]. So, the phenomenon that four proteins involved in beta oxidation were up-regulated suggests that there might be a protective mechanism to keep hepatocytes healthy in early stage PBC, and it might be the stress response to poly I:C and inflammation.

Among the down-regulated proteins, steroid 17-alpha-hydroxylase/17,20 lyase, also known as cytochrome P450 17A1 (CYP17A1), is a single enzyme with both 17α-hydroxylase and 17,20-lyase activities. It is the key rate-limiting enzyme involved in the initial steps of steroidogenesis [32]. CYP17A1 is related with liver toxicities caused by its product, 17-αhydroxysteroids [33]. Down-regulation of CYP17A1 in our PBC mouse model might be the stress response at early stage of PBC to decrease the toxicity to the cell. In addition, sterol 26-hydroxylase (CYP27A) is an important enzyme, not only in the formation of bile acids from cholesterol intermediates in the liver but also in the removal of cholesterol by side chain hydroxylation in extrahepatic tissues [34]. Experiments with bile duct-ligated hamsters showed that bile acid biosynthetic pathway via mitochondrial CYP27A was preferentially inhibited [35]. PBC is a cholestasis liver disease. Mouse model used in this study is in early stages, down-regulated expression of CYP27A might be a predictor for the destruction of bile duct.

Finally, it should point out that our findings are mainly based on iTRAQ analysis. Although the successful validation of 3 differential proteins by Western blotting greatly increases our confidence in iTRAQ results, the certainty of the other 26 differential proteins still remain to be validated once specific antibododies are available.

## Conclusions

In conclusion, through the comparative proteomics study on the liver mitochondria of PBC mouse model, we identified 29 differentially expressed proteins. Most are involved in lipid metabolism and oxidation reduction, and some are involved in the biosynthesis of steroid hormone and primary bile acid. All four differentially expressed proteins involved in fatty acid beta oxidation and most proteins associated with oxidation reductive were up-regulated. Our results provide some insights for the mechanism of pathogenesis in early stage of PBC.

## Competing interests

The authors declare that they have no competing interests.

## Authors’ contributions

GS. Conception and design of the project; purification of liver mitochondria, protein exaction and digestion, analysis and interpretation of iTRAQ data and validation by western blotting; drafting and revising the article critically. CH. Conception and design of the project; construction of mouse models; histopathological staining and analysis, and revising the article critically. HZ &LZ. Acquisition of iTRAQ data, polyclonal antibodies preparation and revising the article. XL and RZ. Construction of mouse models and revising the article. FZ & XZ. Conception of the project; diagnosis of PBC for the animal model and revision of the article. XZ. Fund support. LW&YL. Fund support, conception and design of the whole work, revising the article critically for important intellectual content. LW. analysis and interpretation of data. All of the authors contributed to the final approval of the version published.

## Pre-publication history

The pre-publication history for this paper can be accessed here:

http://www.biomedcentral.com/1471-230X/13/64/prepub

## Supplementary Material

Additional file 1: Table S1 The ratio of individual peptide signals for the 9 down-regulated proteins. **Table S2.** The ratio of individual peptide signals for the 20 up-regulated proteins. Click here for file
